# The National Convention

**Published:** 1846-05

**Authors:** 


### THE NATIONAL CONVENTION.

From all the indications of the day it is now pretty evident, that the proposed medical convention in New York will not be *national.* We have stated heretofore, that the University of Pennsylvania and the Medical Institute of Louisville will not be represented in it.—We learn that the Ohio Medical College, and the St. Louis and Boston medical schools also decline sending delegates. From the tone of Professor Paine's valedictory lecture it is quite certain the University of New York will not take part in it. All this is to be regretted. A convention in the right spirit, national in feeling and aim, composed of representatives from every part of the country, would be felt in its elevating influences by the profession in all the length and breadth of the land. But we begin to doubt whether the eyes of any now living will ever be gladdened by the sight of such a convention.

